# A Rare Case of Thoracoabdominal Paraganglioma: A Case Report and Literature Review

**DOI:** 10.7759/cureus.32504

**Published:** 2022-12-14

**Authors:** Patrícia Baptista, Vânia Benido Silva, Ana Rita Cruz, Liliana Fonseca, Isabel Palma

**Affiliations:** 1 Internal Medicine, Centro Hospitalar Universitário do Porto, Porto, PRT; 2 Endocrinology, Centro Hospitalar Universitário do Porto, Porto, PRT

**Keywords:** metastatic paragangliomas, succinate dehydrogenase b deficiency, catecholamine hypersecretion, sympathetic paraganglioma, paraganglioma

## Abstract

Pheochromocytomas and paragangliomas are rare neuroendocrine tumors. Pheochromocytomas are derived from chromaffin cells of the adrenal medulla, while paragangliomas arise from the extra-adrenal autonomic paraganglia. Paragangliomas can derive from either parasympathetic or sympathetic paraganglia. The majority of parasympathetic ganglia-derived paragangliomas are nonfunctional and symptoms arise from mass effect, while sympathetic paragangliomas are frequently functional and present with symptoms that result from catecholamine hypersecretion. Here, we present the case of a 19-year-old female with hypertension whose biochemical tests revealed elevated plasma and urinary levels of norepinephrine and normetanephrine. Imaging studies showed a left paravertebral mass which was surgically removed. Histopathology confirmed a paraganglioma. Total surgical resection remains the gold-standard treatment and a cure can be achieved; however, all tumors may harbor malignant potential, and a long-term biochemical and imaging follow-up is required in all patients. Screening for genetic germline mutations may be helpful in identifying patients with a higher risk of recurrence or of developing other primary tumors.

## Introduction

Pheochromocytoma and paraganglioma are rare neuroendocrine tumors [1]. The former arises from chromaffin cells of the adrenal medulla, while the latter arises from the extra-adrenal autonomic paraganglia [1,2]. Although these two tumor types are indistinguishable by histologic findings, the anatomical location helps to differentiate between them [3]. The specific incidence of paraganglioma is largely unknown because they are commonly described together with pheochromocytoma [3,4]. The combined estimated annual incidence is approximately 0.8 per 100,000 person-years [4]. A recent nationwide study performed in the Netherlands demonstrated an age-standardized incidence rate of 0.46 and 0.11 per 100,000 person-years for pheochromocytoma and paraganglioma, respectively [5]. Paragangliomas can derive from either parasympathetic or sympathetic paraganglia [4,6]. Although they cannot be differentiated based on histologic findings, parasympathetic and sympathetic paragangliomas are distinct in their anatomic distribution, clinical features, and frequency of an underlying genetic syndrome [4,6]. The majority of parasympathetic ganglia-derived paragangliomas are located in the neck and skull base along the branches of the glossopharyngeal and vagus nerves [4,6,7]. Carotid tumors are the most common, followed by jugulotympanic and vagal paraganglioma [4,6,7]. Most often (in 80-90% of the cases), these tumors are nonfunctional and symptoms arise from mass effect [4,6,7]. Around 75% of sympathetic paragangliomas arise in the abdomen, most frequently at the junction of the vena cava and left renal vein or at the organ of Zuckerkandl (located at the aortic bifurcation or at the origin of the inferior mesenteric artery), about 10% arise at the thorax, and 10% at the bladder and prostate [4,6,7]. The majority of sympathetic paragangliomas are functional and present with symptoms that result from catecholamine hypersecretion, almost always norepinephrine [4,6,7].

About one-third to one-half of paragangliomas can be associated with an inherited syndrome [4,8]. Most paragangliomas are benign [3]. There are no prognostic markers that precisely predict the malignant behavior of these tumors, and only metastases are proof of malignant paraganglioma [3]. The updated 2017 version of the World Health Organization (WHO) classification replaced the term “malignant” and “benign” with “metastatic” and “non-metastatic,” respectively [2,3]. Histological scoring systems, such as the Pheochromocytoma of the Adrenal Gland Score (PASS) and the Grading system for Adrenal Pheochromocytoma and Paraganglioma (GAPP), are frequently used to assess the risk of future dissemination [9,10]. However, few studies have verified and validated these scores, and their reproducibility is still up for debate [9,10]. PASS score was developed in 2002 and was originally designed for pheochromocytomas. It ranges from 0 to 20 points and incorporates 12 different histology-based parameters, namely, the presence of nuclear hyperchromasia, profound nuclear pleomorphism, capsular invasion or vascular invasion, large nests/compact growth, tumor necrosis, high cellularity, cellular monotony, tumor cell spindling, more than three mitoses per 10 high-power fields, atypical mitosis, and extension to the surrounding adipose tissue [9-11]. GAPP score was published in 2014 and incorporates pheochromocytomas and abdominal paragangliomas, along with histological features (such as growth pattern, cellularity, presence of coagulation necrosis, capsular and vascular invasion), biochemical parameters (type of catecholamine production), and immunohistochemical parameters (Ki-67 proliferation index) [9,10,12]. GAPP score ranges from 0 to 10, and paragangliomas can be graded as either well differentiated (0-2 points), moderately differentiated (3-6 points), or poorly differentiated (7-10 points) [9,10,12].

Patients can present with signs and symptoms related to catecholamine hypersecretion or mass effect or may be asymptomatic (detected by incidental findings in computed tomography (CT) or as a carrier of disease-causing mutation) [1,3,4]. The diagnosis can be made by a combination of clinical features, biochemical tests (mainly in a secretory paraganglioma), and imaging (contrast-enhanced CT or magnetic resonance imaging (MRI)) [1,3,4]. Functional imaging (as positron-emission tomography (PET) with ^68^Ga DOTATE and gallium ^68^Ga DOTATOC) has emerged in the last years and may improve the detection and staging of a variety of neuroendocrine tumors, including paraganglioma [13].

## Case presentation

A 19-year-old female with no significant medical history besides being overweight was referred to an internal medicine appointment for investigation of secondary causes of high blood pressure. The patient was diagnosed with hypertension following an episode of severe headache that led to a visit to the emergency department. She denied blurry vision, palpitations, excessive diaphoresis, a recent change in weight, heat or cold intolerance, or muscle weakness. There was no family history of early-onset hypertension, early-onset stroke, or sudden death. Blood pressure was under control with a calcium channel blocker. Laboratory studies revealed elevated serum norepinephrine (11 times above the upper normal limit) and normetanephrine (12 times above the upper normal limit), while the remaining serum analysis was within normal levels (Table 1). Complete blood count, electrolytes, urea, creatinine, and basic metabolic panel were also within normal levels. The patient was not taking any other medication besides the calcium channel blocker at the time of the blood test. Twenty-four-hour urine collection revealed elevated normetanephrine and norepinephrine levels (four and 12 times above the upper normal limit, respectively) (Table 1).

**Table 1:** Initial workup and early postoperative study. TSH = thyroid-stimulating hormone; FT4 = free thyroxine; PTH = parathyroid hormone; ACTH = adrenocorticotropic hormone

	Initial workup	Early postoperative	Reference value
TSH (µUI/mL)	2.77	-	0.51–4.30
FT4 (ng/dL)	1.19	-	0.98–1.63
PTH (pg/mL)	65	-	15–65
Serum calcium (mmol/L)	2.46	-	2.10–2.55
Serum phosphate (mmol/L)	1.30	-	0.87–1.45
Plasma metanephrine (pmol/L)	154	240	<456
Plasma normetanephrine (pmol/L)	12,531	443	<982
Plasma epinephrine (pmol/L)	425	-	20–450
Plasma norepinephrine (pmol/L)	32,282	-	470–2,950
24-hour urine normetanephrine (nmol/d)	9,880	-	480–2,424
24-hour urine norepinephrine (nmol/d)	5,851	-	89–473
Plasma aldosterone (pg/mL)	376	-	97–626
Plasma renin (pg/mL)	26	-	
ACTH (pg/mL)	9.7	-	9–52
Plasma cortisol (µg/dL)	11.4	-	6.2–19.4
Calcitonin (pg/mL)	<2	-	0–20

**Figure 1:**
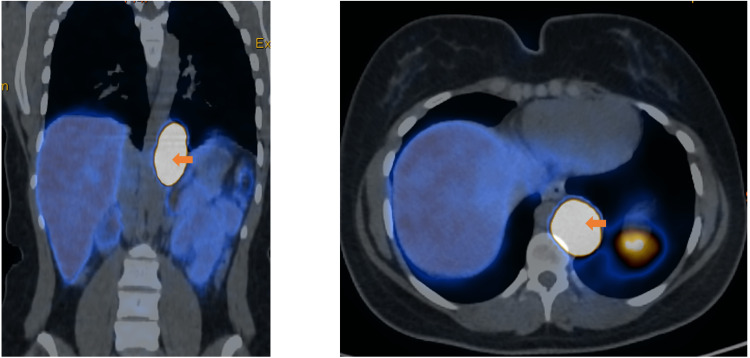
Coronal section and cross-section 68Ga-labeled (DOTA-TOC) positron emission tomography scan showing a left paravertebral mass at the D9-D11 level with abnormal overexpression of somatostatin receptors in activity (arrows).

Contrast-enhanced CT showed a 4.6 × 3.6 × 7.5 cm vascular left paravertebral and para-aortic mass in the thoracoabdominal transition suggestive of a paraganglioma. A ^68^Ga-labeled (DOTA-TOC) PET scan revealed a left paravertebral mass at the D9-D11 level with abnormal overexpression of somatostatin receptors in activity, without other areas of anomalous expression of somatostatin receptors (Figure 1).

The patient initiated adrenergic blockage with phenoxybenzamine, and, a week later, initiated propranolol. Phenoxybenzamine was titrated and increased up to 20 mg three times a day, and propranolol was increased up to 10 mg four times a day. The patient underwent surgical resection of the paravertebral mass without any complications. Intraoperatively, a well-encapsulated 75 × 45 × 40 mm tumor was seen in the left paravertebral region. The pathological examination specimen demonstrated encapsulated tumor composed of diffuse large nests, high cellularity, rounded cells with eosinophilic cytoplasm, and regular centralized nuclei. The proliferative index, evaluated by the expression of Ki67, was 1-3%. Immunohistochemistry features were consistent with neuroendocrine tumor; expression of chromogranin and synaptophysin, multifocal and weak expression of GATA-3, rare sustentacular cells expressing S100 protein, and no expression of succinate dehydrogenase B (SDHB). The histopathology and immunohistochemistry findings confirmed that the tumor was a paraganglioma with SDHB deficiency. A genetic test detected a germline mutation in the *SDHB *gene (c.73-8A>G), in heterozygosity, of uncertain significance. The clinicopathologic tumor staging, by the American Joint Committee on Cancer 8th edition, was pT2 N0 M0.

Following the surgery, her blood pressure remained within the normal range without any antihypertensive medication until discharge. Postoperative serum metanephrine and normetanephrine were within normal values (Table 1). At the time of submission of this paper, the patient is in her second month of follow-up after surgery and remains asymptomatic and normotensive. The patient’s family members are waiting for the result of genetic screening for mutations in the *SDHB *gene.

## Discussion

We present the case of a 19-year-old female with hypertension whose biochemical tests revealed elevated plasma and urinary levels of norepinephrine and normetanephrine. Contrast-enhanced CT scan and ^68^Ga-labeled (DOTA-TOC) PET scan showed a left paravertebral mass with abnormal overexpression of somatostatin receptors. The tumor was surgically removed and a paraganglioma was confirmed by histopathology. The genetic test detected a germline mutation in the *SDHB *gene (c.73-8A>G) of uncertain significance, but the immunohistochemistry of the tumoral tissue detected an absence of SDHB expression, which confirmed the functional consequence of the germline mutation detected. Although the molecular pathogenesis of paraganglioma is incompletely understood, there is some data to support the involvement of hypoxia-inducible factor (HIF), a transcription factor that regulates the expression of genes responsible for angiogenesis, cell proliferation, metastasis, and cell dedifferentiation [4,14,15]. The succinate dehydrogenase is composed of four subunits (A, B, C, and D) and converts succinate to fumarate in the Krebs cycle [14,15]. When a mutation in the B subunit is present this conversion is disrupted leading to a buildup of succinate [14,15]. The excess succinate subsequently inhibits prolyl hydroxylase which stabilizes the HIF [14,15]. *SDH *mutations are seen in several family clusters of paraganglioma, which are described and defined as paragangliomas syndrome one (PGL1) through five (PGL5) depending on the mutated subunit of *SDH *[3,4,14]. PGL4 is associated with pathogenic variants in the *SDHB* gene and is the second most common type of familial paraganglioma [3,4,14]. These patients typically present with an abdominal, pelvic, or thoracic catecholamine-secreting tumor, commonly secreting norepinephrine [4,14]. Due to the role of *SDHB *as a tumor suppressor, patients with *SDHB *pathogenic variants are associated with a higher rate of metastasis, risk of recurrence, or development of other primary tumors [3,4,14,15]. A metastatic workup with functional imaging (such as ^68^Ga-labeled (DOTA-TOC) PET) is recommended at the time of diagnosis. If no metastatic disease is found, lifelong clinical (metanephrine and normetanephrine monitoring) and imaging follow-up is recommended. MRI should be the choice to avoid cumulative radiation exposure [14,16]. This was also the recommended follow-up approach for this patient.

Our patient had a germline mutation in the *SDHB *gene of uncertain significance but the immunohistochemistry confirmed absent SDHB expression, which may indicate that the patient had an increased risk of recurrence and metastatic disease. However, further clinical cases with this germline mutation are needed to confirm our suspicion, along with the follow-up of our patient and genetic studies of the patient’s family members.

As mentioned before, there are no prognostic markers that precisely predict the metastatic potential of paragangliomas, and histological scoring systems, such as the PASS and GAPP scores, may help assess the risk of future dissemination [3,9,10]. In the original cohort, a PASS score of four points or more indicated an increased risk of future aggressive behavior [9-11]. In the original study of the GAPP score, all patients graded as well-differentiated were metastatic-free, while the moderately and poorly differentiated groups had a higher proportion of metastatic cases and lower disease-specific survival [12]. Wachtel et al. conducted a cohort study with 143 subjects to compare the PASS and GAPP scores and concluded that the PASS score had a significant interobserver variability and that a higher GAPP score had a stronger association with aggressive paraganglioma/pheochromocytoma [10]. Our patient had a GASS score of 5 points and a PASS score of 4 points, which indicated that the patient had a higher risk of future aggressive behavior/metastatic disease.

## Conclusions

Functional paragangliomas are a rare and potentially curable cause of hypertension. The clinical, biochemical, radiologic, and morphological features of paragangliomas are important to establish a management plan for treatment and follow-up. Total surgical resection remains the gold-standard treatment. However as all paragangliomas may present the risk of future aggressive/metastatic disease, a long-term follow-up is required in all patients. The genetic study is important as it may detect a germline mutation with implications in the treatment, follow-up, and diagnosis of associated tumors and may have implications for future generations.

Despite the limitations of the current scoring systems, they may help us identify patients with a higher risk of future metastatic disease. Probably the best score is yet to come and will be the one that combines histopathology, immunohistochemical and clinical data, and germline and somatic tumor markers.
